# Supplementary material for: FERN – a Java framework for stochastic simulation and evaluation of reaction networks
Source: BMC Bioinformatics. 2008 Aug 29;9:356. doi: 10.1186/1471-2105-9-356 (PMC2553347; doi:10.1186/1471-2105-9-356)
Supplement: Additional file 1 — FERN distribution, Version 1.3. This archive contains the FERN source code and binaries as well as documentation and example models in FernML and SBML. [file 1471-2105-9-356-S1.zip › fern/doc/javadoc/fern/simulation/algorithm/package-use.html]

Uses of Package fern.simulation.algorithm


---


|  |  |  |
| --- | --- | --- |
| **Overview** \| **Package** \| Class \| **Use** \| **Tree** \| **Deprecated** \| **Index** \| **Help** | |  |
| PREV   NEXT | **FRAMES**    **NO FRAMES**     **All Classes** |  |


---


## **Uses of Package fern.simulation.algorithm**

| Packages that use fern.simulation.algorithm | |
| --- | --- |
| **fern.simulation.algorithm** | Provides algorithms for stochastic simultion of reaction networks. |

Classes in fern.simulation.algorithm used by fern.simulation.algorithm

****AbstractBaseTauLeaping****
  
          Base class for all tau leaping procedures (which are different in the methods choosing
the timestep candidates for critical and noncritical reactions).

****AbstractTauLeapingPropensityBoundSimulator****
  
          There are some possibilities to bind the expected change of the propensities by a
value epsilon in order to fulfill the leap condition.

****GillespieEnhanced****
  
          This is an enhanced version of the original Direct method developed by
Gillespie.

---


|  |  |  |
| --- | --- | --- |
| **Overview** \| **Package** \| Class \| **Use** \| **Tree** \| **Deprecated** \| **Index** \| **Help** | |  |
| PREV   NEXT | **FRAMES**    **NO FRAMES**     **All Classes** |  |


---
